# A Model-Based Optimization Framework for Iterative Digital Breast Tomosynthesis Image Reconstruction

**DOI:** 10.3390/jimaging7020036

**Published:** 2021-02-13

**Authors:** Elena Loli Piccolomini, Elena Morotti

**Affiliations:** Department of Computer Science and Engineering, University of Bologna, Via Mura Anteo Zamboni 7, 40126 Bologna, Italy; elena.morotti4@unibo.it

**Keywords:** Digital Breast Tomosynthesis, few-views tomography, model-based method, Iterative Reconstruction algorithm, Total Variation regularization

## Abstract

Digital Breast Tomosynthesis is an X-ray imaging technique that allows a volumetric reconstruction of the breast, from a small number of low-dose two-dimensional projections. Although it is already used in the clinical setting, enhancing the quality of the recovered images is still a subject of research. The aim of this paper was to propose and compare, in a general optimization framework, three slightly different models and corresponding accurate iterative algorithms for Digital Breast Tomosynthesis image reconstruction, characterized by a convergent behavior. The suggested model-based implementations are specifically aligned to Digital Breast Tomosynthesis clinical requirements and take advantage of a Total Variation regularizer. We also tune a fully-automatic strategy to set a proper regularization parameter. We assess our proposals on real data, acquired from a breast accreditation phantom and a clinical case. The results confirm the effectiveness of the presented framework in reconstructing breast volumes, with particular focus on the masses and microcalcifications, in few iterations and in enhancing the image quality in a prolonged execution.

## 1. Introduction

Digital Breast Tomosynthesis (DBT) is a 3D X-ray cone-beam Computed Tomography (CT) technique for the early detection of breast tumors [1,2].

While the traditional digital mammography provides a unique 2D breast image, DBT reconstructs the breast as a stack of 2D images by using a comparable radiation dose. Hence, DBT is also used in screening programs, because the volumetric reconstruction reduces the tissue overlaps allowing for a better visibility of malignant structures. DBT is characterized by a limited-angle geometry: since the object is scanned only from a narrow angular range, the DBT projection data are incomplete if compared to classical CT cases.

The reconstruction algorithm plays an important role, influencing the accuracy of the recovered breast images. It is well known that traditional fast analytic reconstruction methods, such as Feldkamp [3], produce poor noisy images in limited-angle tomography, hence they have been left in favor of Iterative Reconstruction (IR) algorithms [4,5,6]. IR solvers provide a sequence of solutions, by computing an improved reconstructed volume at each iteration. Iterative approaches have been introduced since the first years of CT, but they have not been used for a long time due to their high computational time request. Recently, IR methods got a renewed interest in scientific communities and among the major vendors, due to the advent of more performing processors [7]. As a consequence, a wide amount of IR methods has been proposed to reconstruct tomographic images and an exhaustive analysis can be found in [8], whereas an overview of IR methods for CT image reconstruction is presented in Section 1.1.

In this work we consider three IR algorithms as solvers of three slightly different model-based formulations. They all solve optimization problems where the objective function both describes the CT process (by modeling the physics of the system and including the presence of noise on the projection data) and introduces some image priors. Such a mathematical approach is quite uncommon in 3D tomographic imaging, where a constrained formulation is preferred [9,10,11,12]. In particular, we mainly consider the objective function as the sum of the Least Squares (LS) data-fitting term and the Total Variation (TV) regularization function. The TV regularizer is chosen by many authors because of its excellent shape recovering and denoising properties, even if it is known that TV can produce staircasing effects when the regularization parameter is too high [8,9,10,11,12,13]: the choice of the regularization parameter plays a fundamental role in the considered framework.

**Figure 1:**
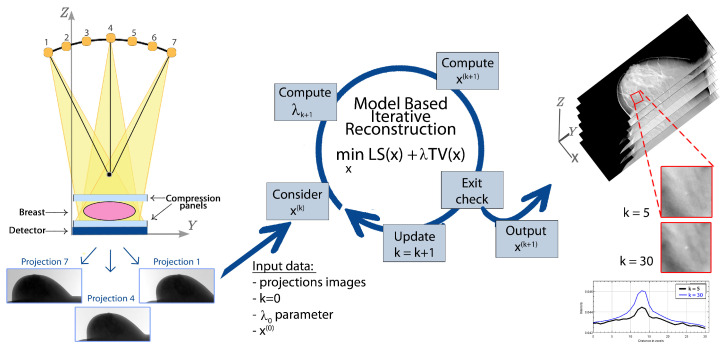
Scheme of the Digital Breast Tomosynthesis (DBT) reconstruction process. On the left, a draft of the frontal (coronal) section of a DBT system acquiring projection images of the breast; in the center, a chart representing the *k*th iteration of the algorithm computing the sequence ${\left\{x^{\left(k\right)}\right\}}_{k}$ of approximate solutions by solving the model-based minimization problem; on the right, the evaluation of reconstructed volumes by inspection of cancer objects of interest.

Figure 1 shows the model-based approach workflow, from the numerical modeling of the projection step during the breast scanning, to the reconstructed volume inspection searching for breast cancer objects, via the implementation of an iterative solver for the minimization problem.

### 1.1. A Short Review on Iterative Methods for CT

The worldwide increasing interest in Compressive Sensing (CS) [14] has promoted the novel model-based iterative approach (which uses an optimization framework to exploit CS theory). Among the wide class of model-based IR procedures, the so called Sparsity-Exploiting Image Reconstruction (SEIR) methods have produced significant improvement to the image quality in all the low-dose CT applications (see [8,13] and references therein). In particular, many authors have introduced the TV regularization function to take advantage of the sparsity in the image gradient domain for edge detection [4,5,15,16,17,18,19,20,21,22]. This property turns into practice as a noise smoothing effect and as a reliable detection of shape and size of anatomical objects.

It is possible to distinguish two main categories of algorithms in the class of SEIR methods: the approximate solvers and the accurate solvers. The first one contains algorithms using, at each step, an algebraic approach (such as SART and SIRT) and then decreasing the TV of the just calculated solution. Examples are the well-known POCS algorithm and its developments [16,23,24]. They provide reliable reconstructions in few iterations, but the quality of the recovered images strongly depends on the tuning of many inner parameters and the algorithm convergence is not guaranteed. On the other hand, the accurate solvers are optimization methods minimizing an objective function, which is defined as a sum of a data-fitting term and a regularization function. The two quantities are typically weighted by a regularization parameter. This class is represented by classical optimization methods adapted to the huge size 3D tomographic reconstruction problems. Their solution is proved to converge to the exact solution of the minimization problem.

Nowadays, only preliminary investigations on simulations or phantoms have been performed to analyze the results of accurate solvers for few-views CT applications [25,26,27].

Concerning existing rules for the regularization parameter choice in tomography, in [28] the authors propose a strategy based on multiresolution and apply it to 2D reconstructions. The proposed rule is very promising, but it is quite expensive for a very large size 3D application, such as DBT image reconstruction. An exhaustive list of existing rules for the selection of the regularization parameter is reported in [28].

### 1.2. Aim and Contribution of the Paper

The aim of this paper was to propose a new TV-based optimization framework for the reconstruction of DBT images. The framework, described in a rigorous numerical setting, includes both constrained and unconstrained models, thus it is a flexible tool easily enabling the use of different data-fitting or regularization terms as well as the addition of further box constraints, to reconstruct reliable volumes from subsampled noisy data.We are also interested in finding an automatic strategy to set the regularization parameter, which strongly affects the quality of the reconstructions, in order to avoid its manually tuning (which is infeasible in a clinical setting).

The contribution of this work can be summarized as follows.

- We propose three different models inside a unique optimization framework, combining a data-fitting function (identified in the least squares function) and a regularization TV-like term. We solve the minimization problem with efficient iterative algorithms, converging to the global minimum of the problem, and we analyze and compare the results achieved from real noisy and large-size data sets. Two of these models have been presented in our previous works on low-sampled CT image reconstruction [26,27], but they were applied to small simulated data sets with different geometries (not limited angles). This optimization approach is new for DBT applications.
- We propose a user independent and computationally effortless rule to set and adapt the regularization parameter at each iteration of the algorithms.
- In order to assess our proposals, we implement the methods and test them on real large-size and noisy projection data from both a breast accreditation phantom and a human patient. We analyze the algorithms performance in recovering the breast tumor objects of interest (i.e., masses and microcalcifications clusters), by means of measures of merits and visual inspection, at different stages of the Iterative Reconstruction process. We analyze the volume via its recovered slices, both perpendicularly and along the *Z* direction (see Figure 1).

We remark that in clinical routine almost real time reconstructions are required. However, we underline the importance of improving the image quality with ongoing iterations in longer execution times, for two main reasons: first, having more reliable images can be crucial in difficult diagnosable cases to avoid false interpretations; second, the fast evolution of multiprocessor boards, such as GPUs, is drastically reducing the time per iteration of the methods, hence we can suppose that more iterations could be performed in clinical reconstructions in a next future.

The paper is organized as follows. In Section 2, we state the optimization framework for the image reconstruction, thus we illustrate the three proposed model-based approach and IR solvers in Section 3. Section 4 and Section 5 present the data sets and the experimental results, respectively. Finally, Section 6 contains some conclusions.

## 2. The Optimization Framework in a Model-Based Formulation

Mathematically, tomographic image reconstruction is an ill-posed inverse problem whose solution can be obtained by minimizing a suitable objective function related to the physical process. To define the model describing image reconstruction, a deep understanding of the acquisition steps characterizing the DBT technique is therefore crucial. A schematic example of a DBT system is shown in the left of Figure 1. In DBT routine, the breast is first compressed along the *Z*-axis, over the flat detector plane. The source moves along an arc trajectory and emits low-dose radiations from a discrete number of angles. Once the X-ray cone-beam has passed through the body, the detector records its attenuation: the set of the resulting projection images constitutes the raw tomographic data set. The breast volume to be recovered is composed by a stack of high resolution images, parallel to the detector plane along the *Z* vertical direction.

In order to define the numerical model of tomographic image formation, we recall the physical law underlying the acquisition process. Supposing the 2D detector panel is made of $N_{p}$ recording units, for each fixed projection angle θ and *i*th recording unit, the Lambert–Beer law relates the projection $P_{i}^{\theta }$, along a ray $R^{\theta }$, to  the attenuation coefficient function μ(w) of a point *w* [29] as:

(1) $$\int_{R^{\theta }}\mu \left(w\right)dR=-ln\left(\frac{P_{i}^{\theta }}{P_{0}}\right),\;\;i=1,\ldots ,N_{p},$$

where $P_{0}$ represents the intensity of the energy emitted by the X-ray source. By discretizing the 3D object into $N_{v}$ voxels and by considering all the $N_{\theta }$ scanning angles, the integral in (1) yields the following linear system:

(2) Mx=b.

In Equation (2), we denote with *x* the $N_{v}$ dimensional vector stacking the attenuation coefficients of all the voxels, while *b* is the vector of size $N_{d}=N_{p}\cdot N_{\theta }$ storing all the projections (i.e., the right hand sides of (1)) and *M* is the matrix of size $N_{d}\times N_{v}$, built according to the DBT device geometry and representing the projection process onto the detector.

Some issues arise when solving the linear system (2) as an inverse problem, such as the existence of infinite solutions (since $N_{v}>N_{d}$) and the presence of high noise in the reconstructed images (due to the ill-posedness of the problem). The model-based approach is introduced to overcome these numerical controversies, by adding some a priori information. The resulting formulation can be stated as an unconstrained or constrained minimization problem [8], involving a data-fitting function J(x) and a prior operator R(x) (acting here as a regularizer).

In particular, we settle J(x) as the Least Squares (LS) function

(3) $$LS\left(x\right)={∥Mx-b∥}_{2}^{2}$$

and R(x) as the Total Variation (TV) operator defined as [30]:

(4) $$TV\left(x\right)=\sum_{j=1}^{N_{v}}{∥\nabla x_{j}∥}_{2}.$$

The TV operator is not differentiable in the origin, thus we consider the smoothed TV version:

(5) $$TV_{\beta }\left(x\right)=\sum_{j=1}^{N_{v}}\sqrt{∥\nabla x_{j}{∥}_{2}^{2}+\beta^{2}}$$

where β is a small positive parameter [30], when a solver requires the objective function differentiability.

We remark that both the TV and the $TV_{\beta }$ quantities can be computed with forward differences with boundary periodic conditions on the volumes. In addition, if we reshape the vector *x* of length $N_{v}$ as a 3-dimensional object x of size $N_{x}\times N_{y}\times N_{z}$, we can introduce the notation $j_{x},j_{y},j_{z}$ to indicate the indices of the *j*th voxel with respect to the three Cartesian axes ($j_{x}=1,\ldots ,N_{x},\;j_{y}=1,\ldots ,N_{y}$ and $j_{z}=1,\ldots ,N_{z}$). Hence, for all $j=1,\ldots ,N_{v}$ the $∥\nabla x_{j}{∥}_{2}^{2}$ elements in Equations (4) and (5) can be read as $∥\nabla x_{j}{∥}_{2}^{2}={∥\nabla x_{j_{x},j_{y},j_{z}}∥}_{2}^{2}$ and thus expressed as:

(6) $$∥\nabla x_{j_{x},j_{y},j_{z}}{∥}_{2}^{2}={\left(x_{j_{x}+1,j_{y},j_{z}}-x_{j_{x},j_{y},j_{z}}\right)}^{2}+{\left(x_{j_{x},j_{y}+1,j_{z}}-x_{j_{x},j_{y},j_{z}}\right)}^{2}+{\left(x_{j_{x},j_{y},j_{z}+1}-x_{j_{x},j_{y},j_{z}}\right)}^{2}.$$

Since the model-based problem statement is a flexible framework, in this work we consider three different LS-TV like formulations, i.e.,:

(7) $$\underset{x\geq 0}{arg min}f\left(x\right)=LS\left(x\right)+\lambda TV_{\beta }\left(x\right).$$

(8) $$\underset{x}{arg min}f\left(x\right)=LS\left(x\right)+\lambda TV_{\beta }\left(x\right)$$

(9) $$\underset{x}{arg min}TV\left(x\right)\;s.t.\;LS\left(x\right)\leq \epsilon^{2},x\geq 0.$$

where λ≥0 is a regularization parameter and ϵ≥0 is an estimate of a noise measure. The introduction of the box constraint x≥0 reflects the non-negativity property of the linear attenuation coefficient μ in (1), hence it is coherent to the DBT numerical formulation.

We remark that exploiting the linearity of the objective function f(x) in (7) and (8), the objective function gradient $\nabla f\left(x\right)=\nabla LS\left(x\right)+\lambda \nabla TV_{\beta }\left(x\right)$ can be evaluated by separately computing ∇LS(x) as:

(10) $$\nabla LS\left(x\right)=2\left(M^{T}Mx-M^{T}b\right)$$

and $\nabla TV_{\beta }\left(x\right)$ through finite forward differences (see [27] for its detailed derivation).

## 3. Iterative Optimization Methods

For each considered model formulation (7)–(9), we propose an accurate solver whose convergence to the global minimum is demonstrated. We thus apply and compare the Scaled Gradient Projection (SGP), the Fixed Point (FP) and the Chambolle–Pock (CP) methods to solve the optimization problems (7), (8) and (9), respectively.

Among the wide class of optimization methods they have been chosen since they satisfy the requirements necessary to be usable on DBT devices:

- a fast error decreasing in the initial algorithm execution, in order to obtain a good image in few iterations;
- a low computational cost per iteration (which is mainly determined by the number of matrix-vector products), to efficiently run the solver in short time;
- a limited request of memory, to solve real-size problems on commercially affordable hardware.

A challenging issue (common to the implementation of the three algorithms) is the computation of the projection matrix *M*; since it can not be stored due to its huge dimensions, it must be recalculated at each call. Thus, this section ends with a focus on the algorithm we use to generate *M*.

### 3.1. Scaled Gradient Projection Algorithm

The SGP algorithm is a first order accelerated method. We apply it to solve the non-negative constrained and differentiable optimization problem (7). Algorithm 1 reports the main steps of the SGP algorithm.

At each *k*th iteration, the new solution is computed by moving along a descent direction $d^{\left(k\right)}$ of a quantity $\eta_{k}>0$, as:

(11) $$x^{\left(k+1\right)}=x^{\left(k\right)}+\eta_{k}d^{\left(k\right)}.$$

The direction $d^{\left(k\right)}$ is obtained through a projection $P_{+}$ onto the non-negative orthant of the scaled gradient direction $-S_{k}\nabla f(x^{\left(k\right)}$:

(12) $$d^{\left(k\right)}=P_{+}\left(\;x^{\left(k\right)}-\alpha_{k}S_{k}\nabla f\left(x^{\left(k\right)}\right)\right)-x^{\left(k\right)}$$

where $\alpha_{k}$ is the step length and $S_{k}$ is the scaling matrix (step 7 in Algorithm 1).

Essentially, the method follows a Gradient Projection approach accelerated by choosing the $\alpha_{k}$ step length with Barzilai–Borwein techniques as proposed in [27] and by introducing a suitable scaling matrix improving the matrix conditioning. In particular, the scaling matrix $S_{k}$ is a diagonal matrix with entries in a limited interval. To update $S_{k}$ (line 5 of Algorithm 1), we compute a splitting of the objective function gradient into its positive and negative parts, as:

(13) ∇f(x)=V(x)−U(x),

where V(x)>0 and U(x)≥0. The diagonal elements $s_{j,j}^{\left(k\right)}$ of $S_{k}$ are updated, for $j=1,\ldots ,N_{v}$ as:

(14) $$s_{j,j}^{\left(k\right)}=\min\left(\;\rho_{k},\max\left(\;\frac{1}{\rho_{k}},\frac{x_{j}^{\left(k\right)}}{V_{j}\left(x^{\left(k\right)}\right)}\right)\right)$$

where ${\left\{\rho_{k}\right\}}_{k}$ is a decreasing bounded positive sequence, ensuring the procedure convergence [31]. We finally denote by $S_{\rho_{k}}$ the set of diagonal matrices with entries in the interval $\left[\frac{1}{\rho_{k}},\rho_{k}\right]$.

Regarding the convergence, it is proved in [32] that the SGP algorithm converges without any further restriction on the step length $\alpha_{k}$ and on the scaling matrix $S_{k}$ to the unique minimum of (7). In [31], the authors proved that the theoretical convergence rate of the SGP method is O(1/k).
**Algorithm 1** Scaled Gradient Projection algorithm (SGP)**Input:**M,b,λ1:**Initialize:**$x^{\left(0\right)}\geq 0,\;\;\gamma ,\sigma \in \left(0,1\right),\;\;0<\alpha_{min}\leq \alpha_{max},$2:k = 03:**while** not convergence **do**4:    Compute $g^{\left(k\right)}=2\left(M^{T}Mx^{\left(k\right)}-M^{T}b\right)+\lambda \nabla TV_{\beta }\left(x^{\left(k\right)}\right)$5:    Compute $S_{k}\in S_{\rho_{k}}$ as in (14)6:    Define $\alpha_{k}\in \left[\alpha_{min},\alpha_{max}\right]$ with alternate BB rules7:    $d^{\left(k\right)}=P_{+}\left(x^{\left(k\right)}-\alpha_{k}S_{k}g^{\left(k\right)}\right)-x^{\left(k\right)}$8:    $\eta_{k}=1$9:    **while** $f\left(x^{\left(k\right)}+\eta_{k}d^{\left(k\right)}\right)>f\left(x^{\left(k\right)}\right)+\sigma \eta_{k}{\left(g^{\left(k\right)}\right)}^{T}d^{\left(k\right)}$ **do**10:        $\eta_{k}=\gamma \eta_{k}$11:    $x^{\left(k+1\right)}=x^{\left(k\right)}+\eta_{k}d^{\left(k\right)}$12:    k = k+1**Output:**$x^{\left(k\right)}$

### 3.2. The Fixed Point Algorithm

The FP algorithm solves the unconstrained problem (8) and it has been proposed for image deblurring in [33]. Starting from this approach, we derived the lagged diffusivity FP Algorithm 2 for 3D tomographic image reconstruction.
**Algorithm 2** Lagged diffusivity Fixed Point algorithm (FP)**Input:**M,b,λ,maxiter1:**Initialize:**$x^{\left(0\right)}\geq 0$2:**for**k=0 to maxiter−1 **do**3:    Compute $g^{\left(k\right)}=2\left(M^{T}Mx^{\left(k\right)}-M^{T}b\right)+\lambda \nabla TV_{\beta }\left(x^{\left(k\right)}\right)$4:    Solve the linear system $H_{k}d^{\left(k\right)}=-g^{\left(k\right)}$, where $H_{k}=M^{T}M+\lambda L\left(x^{\left(k\right)}\right)$, with the Conjugate Gradient method.5:    $x^{\left(k+1\right)}=x^{\left(k\right)}+d^{\left(k\right)}$**Output:**$P_{+}\left(x^{\left(k+1\right)}\right)$

At each *k*th iteration, the FP algorithm updates the solution with the following rule:

(15) $$x^{\left(k+1\right)}=x^{\left(k\right)}+d^{\left(k\right)}$$

where the descent direction $d^{\left(k\right)}$ is computed by solving a linear system $H_{k}d^{\left(k\right)}=-\nabla f\left(x^{\left(k\right)}\right)$ (line 4 of Algorithm 2). The matrix $H_{k}=M^{T}M+\lambda L\left(x^{\left(k\right)}\right)$ in line 4 approximates the Hessian matrix. It contains the seven diagonals banded matrix $L(x^{\left(k\right)})$ which is the discretization matrix of the diffusion operator L(x) so that L(x)x=∇TV(x) [30]. We solve the linear system with very few iterations of a Conjugate Gradient (CG) algorithm [34]. We stop it far before convergence, both to limit the computational time and to prevent noise from affecting the solution. We remark that each CG iteration requires a matrix-vector product involving $H_{k}$ and that, to save memory space, we perform it without storing the matrix $H_{k}$. We only store $L(x^{\left(k\right)})$ and re-compute *M* and $M^{T}$ at run time. At the end, we project the last computed solution onto the non-negative orthant. For more details on the FP method applied to tomographic image reconstruction and its convergence, see [26] and [35], respectively.

### 3.3. The Chambolle–Pock Algorithm

The CP algorithm (originally proposed in [36]) solves the constrained minimization problem stated as in (9), by considering an equivalent unconstrained formulation:

(16) $$\underset{x}{arg min}F\left(Kx\right)+G\left(x\right)$$

where

(17) $$F\left(Kx\right)=\delta_{B_{2}\left(\epsilon \right)}\left(Mx-b\right)+\lambda TV\left(x\right)\;with\;\lambda >0$$

and the $B_{2}$ ball is the set $B_{2}\left(\epsilon \right)=\{y\in R^{N_{d}}{\mathrm{such}\;\mathrm{that}∥y∥}_{2}\leq \epsilon \}$ and its indicator function $\delta_{B_{2}\left(\epsilon \right)}$ is defined as

(18) $$\delta_{B_{2}\left(\epsilon \right)}\left(y\right)=\left\{\begin{array}{cc} 0 & y\in B_{2}\left(\epsilon \right)\\ \infty & y\notin B_{2}\left(\epsilon \right). \end{array}\right.$$

In addition, we set:

(19) $$G\left(x\right)=\delta_{\Omega }\left(x\right)$$

as the indicator function $\delta_{\Omega }\left(x\right)$ of the convex set $\Omega =\{x\in R^{N_{v}}\mathrm{such}\;\mathrm{that}x\geq 0\}$ to embed the non-negativity constraint in the unconstrained formulation. Equation (17) allows to fully exploit the linearity of the *K* transform, combining here both the X-ray projector M and the discrete gradient operator, if we build *K* as the following four-blocks matrix:

(20) $$K=\left(\begin{array}{c} M\\ \nabla_{x}\\ \nabla_{y}\\ \nabla_{z}. \end{array}\right)$$

where $\nabla_{x},\nabla_{y}$ and $\nabla_{z}$ are the forward difference operators acting along the X,Y and *Z* axes, respectively.

Considering the convex conjugate $F^{*}$ of *F*, defined as $F^{*}\left(y\right)=\max_{x}\left\{x^{T}y-F\left(x\right)\right\}$, and the proximal mappings of $F^{*}$ and *G*, i.e.,

(21) $$\begin{array}{cc} prox_{\sigma }\left[F^{*}\right]\left(y\right) & =\underset{\bar{y}}{arg min}\left\{F^{*}\left(\bar{y}\right)+\frac{1}{2\sigma }{∥y-\bar{y}∥}_{2}^{2}\right\}\\ prox_{\tau }\left[G\right]\left(x\right) & =\underset{\bar{x}}{arg min}\left\{G\left(\bar{x}\right)+\frac{1}{2\tau }{∥x-\bar{x}∥}_{2}^{2}\right\}, \end{array}$$

the *k*th CP iteration can be summarized in the following three steps:

1. compute $y^{\left(k+1\right)}$ as $prox_{\sigma }\left[F^{*}\right]\left(y^{\left(k\right)}+\sigma K{\bar{x}}^{\left(k\right)}\right)$;
2. compute $x^{\left(k+1\right)}$ as $prox_{\tau }\left[G\right]\left(x^{\left(k\right)}-\tau K^{T}y^{\left(k+1\right)}\right)$;
3. define ${\bar{x}}^{\left(k+1\right)}$ with an extrapolation step: ${\bar{x}}^{\left(k+1\right)}=x^{\left(k+1\right)}+\theta \left(x^{\left(k+1\right)}-x^{\left(k\right)}\right)$ and θ>0.

We denote as $\left(\nabla_{x};\nabla_{y};\nabla_{z}\right)$ a matrix of size $3N_{v}\times N_{v}$ and we use Matlab-like notation to indicate element by element matrix multiplication and division. In particular, the proximal mapping $prox_{\sigma }\left[F^{*}\right]$ can be computed as the sum of two independent blocks, as in lines 5–6 and 7–9 of the Algorithm 3; its detailed derivation can be found in [37] for 2D imaging applications. We have here extended the method to 3D imaging. For the sake of clarity, we remark that the weights $w^{\left(k\right)}$, ${\bar{w}}^{\left(k\right)}$ and $\bar{\bar{w^{\left(k\right)}}}$ in lines 7–9 are stored in $3N_{v}$-dimensional variables $w=(w_{1},w_{2},w_{3})$ where $w_{i}\in R^{N_{v}}$ for i=1,2,3, whereas $|\bar{w}|$ is a vector of size $N_{v}$ with components

$$|{\bar{w}}^{\left(k\right)}{|}_{j}={∥\left(\begin{array}{c} {\left({\bar{w}}_{1}^{\left(k\right)}\right)}_{j}\\ {\left({\bar{w}}_{2}^{\left(k\right)}\right)}_{j}\\ {\left({\bar{w}}_{3}^{\left(k\right)}\right)}_{j} \end{array}\right)∥}_{2}^{2}\;\mathrm{for}\;\mathrm{all}j=1,\ldots ,N_{v}.$$

**Algorithm 3** Chambolle–Pock algorithm (CP)**Input:**: M,b,ϵ,maxiter1:**Compute:**Γ as an approximation of ${∥K∥}_{2}$2:**Initialize:**$\tau =\sigma =\frac{1}{\Gamma }>0$, θ∈[0,1]3:**Initialize:**$x^{\left(0\right)}\in R^{N_{v}}$$\left(x^{\left(0\right)}\geq 0\right)$, ${\bar{x}}^{\left(0\right)}\in R^{N_{v}},y^{\left(0\right)}\in R^{N_{d}}$ and $w^{\left(0\right)}\in R^{3\cdot N_{v}}$ to zeros-vectors4:**for**k=0 to maxiter−1 **do**5:    ${\bar{y}}^{\left(k\right)}=y^{\left(k\right)}+\sigma \left(M{\bar{x}}^{\left(k\right)}-b\right)$6:    $y^{\left(k+1\right)}=max(∥{\bar{y}}^{\left(k\right)}{∥}_{2}-\sigma \epsilon ,0)\frac{{\bar{y}}^{\left(k\right)}}{∥{\bar{y}}^{\left(k\right)}{∥}_{2}}$7:    ${\bar{w}}^{\left(k\right)}=w^{\left(k\right)}+\sigma \left(\nabla_{x};\nabla_{y};\nabla_{z}\right){\bar{x}}^{\left(k\right)}$8:    $\bar{\bar{w^{\left(k\right)}}}=\left(\right|{\bar{w}}^{\left(k\right)}\left|,\right|{\bar{w}}^{\left(k\right)}\left|,\right|{\bar{w}}^{\left(k\right)}\left|\right)$9:    $w^{\left(k+1\right)}={\bar{w}}^{\left(k\right)}.*(\lambda ./max\left(\lambda ,\bar{\bar{w^{\left(k\right)}}}\right)$10:    $x^{\left(k+1\right)}=x^{\left(k\right)}-\tau \left(M^{T}y^{\left(k+1\right)}+{\left(\nabla_{x};\nabla_{y};\nabla_{z}\right)}^{T}w^{\left(k+1\right)}\right)$11:    $x^{\left(k+1\right)}=P_{+}\left(x^{\left(k+1\right)}\right)$12:    ${\bar{x}}^{\left(k+1\right)}=x^{\left(k+1\right)}+\theta \left(x^{\left(k+1\right)}-x^{\left(k\right)}\right)$**Output:**$x^{\left(k+1\right)}$

The proximal mapping of *G* is defined as:

(22) $$\begin{array}{cc} prox_{\tau }\left[G\right]\left(x\right) & =\underset{\bar{x}}{arg min}\left\{\delta_{\Omega }\left(x\right)+\frac{1}{2\tau }{∥x-\bar{x}∥}_{2}^{2}\right\}\\ \; & =\underset{\bar{x}\in \Omega }{arg min}\left\{\frac{1}{2\tau }{∥x-\bar{x}∥}_{2}^{2}\right\}\\ \; & =P_{+}\left(x\right) \end{array}$$

hence, it is exactly the projection $P_{+}\left(x\right)$ of *x* onto the feasible set Ω (line 11 of Algorithm 3). The updated iterate $x^{\left(k+1\right)}$ is computed with an extrapolation strategy as in line 12 of Algorithm 3. The algorithm convergence is demonstrated in [36].

We finally observe that the algorithm needs to compute the value Γ (line 1 of Algorithm 3): to estimate the matrix 2-norm as $\Gamma \approx {∥K∥}_{2}=\sqrt{\rho (K^{T}K)}$ (where ρ is the spectral radius of a matrix), we perform two iterations of the power method for computation of the maximum eigenvalue in module [38].

### 3.4. User-Independent Choice of the Regularization Parameter 

In model-based optimization approaches, the choice of the regularization parameter λ plays a key role for the quality of the reconstruction and it represents a crucial challenge in a clinical setting, where the trial-and-error approach is not doable for each reconstruction. Moreover, experimental results show that Hence, we propose to reduce the regularization weight along the iterations, by choosing the λ values with a decreasing updating rule. Interestingly, state-of-art studies have already proposed semi-automatic rules for the selection of a decreasing sequence ${\left\{\lambda_{k}\right\}}_{k},\;k=1,\cdots$ of regularization parameters defining a sequence of minimization problems, whose solutions converge to a good reconstructed image. See [39] for more details and the convergence proof.

We propose the following fully-automatic strategy to compute a decreasing sequence ${\left\{\lambda_{k}\right\}}_{k}$. At the beginning of our algorithm, we leave out the regularization by setting the first parameter $\lambda_{0}=0$: we are in fact interested in a very good data fitting, to recover as many image features as possible. Next, the starting value $\lambda_{1}$ is set to balance the residual norm and the amount of TV of the first iterate. Afterward, we propose to decrease λ of a constant factor 1/k at each *k*th iteration, since we need a very simple and computationally cheap rule, reducing the regularization weight slightly. The resulting strategy is summarized in the following scheme and it can be introduced in each of the previously considered algorithms.

- Set $\lambda_{0}=0$ to initialize the algorithm and run the first iteration (labeled with *k* = 0) to compute $x^{\left(1\right)}$;
- Set $\lambda_{1}=\frac{\sqrt{LS(x^{\left(1\right)})}}{TV(x^{\left(1\right)})}$ and use it to compute $x^{\left(2\right)}$;
- For each k≥2, set
  (23) $$\lambda_{k}=\frac{1}{k}\lambda_{1}$$
  and use it to compute $x^{\left(k+1\right)}$.

### 3.5. The Projection Matrix Algorithm

In addition to the choice of the model parameter and the solver, in optimization approach a key point consists in numerical modeling the geometric projection process, schematically displayed from a frontal view in Figure 1, through a matrix.

The coefficient matrix *M* of the linear system (2) is commonly called *projection operator* in tomography, since it represents the action of the tomographic system in projecting an object onto the detector, whereas the matrix modeling the backprojection of the tomographic data onto a volume is called *backprojection operator*. In the proposed optimization algorithms, the backprojection coincides with the transpose matrix $M^{T}$.

**Figure 2:**
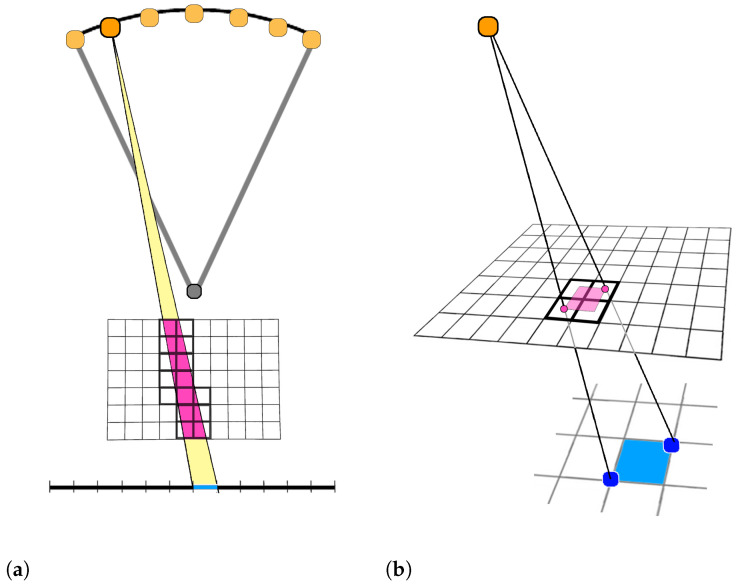
Schematic draw representing the Distance Driven approach to compute the system matrix. (**a**) View on the YZ plane of an X-ray projection onto a single pixel, from a fixed angle. The intersection of the X-ray beam with the volume is highlighted in magenta. (**b**) The magenta area represents the backward projection of the blue recording unit onto a volume slice parallel to the XY plane.

Different algorithms have been proposed in literature for the computation of the matrix *M*. We have adopted the Distance Driven (DD), which accurately models the discretization of the Lambert–Beer’s law (1) for cone-beam projections [40]. In DD, *M* of size $N_{v}\times \left(N_{p}\cdot N_{\theta }\right)$ is constituted by $N_{\theta }$ submatrices $M^{\theta }$ of size $N_{v}\times N_{p}$. Each element $M_{i,j}^{\theta }$ represents the contribution of the *j*th voxel (for $j=1,\ldots N_{v}$) to the projection onto the *i*-th detector pixel (for $i=1,\ldots N_{p}$), for a projection angle θ. Images in Figure 2 help in understanding the DD procedure. In Figure 2a, for a scanning angle, we consider the X-ray cone-beam projecting onto the *i*th blue pixel and intersecting the voxels with bold contours (in the magenta colored area). Only these voxels contribute to the value of the projection in the considered pixel. In Figure 2b, we highlight the *i*th cell of the detector (the blue area) and its backward footprint on a plane parallel to the detector (the magenta area). The ratio between the magenta area inside the *j*th voxel and the whole magenta extension is proportional to the value $M_{i,j}$ of the matrix. For all the voxels *j* not contributing to the *i*th projection, the corresponding matrix element $M_{i,j}=0$; hence, *M* is extremely sparse. However, despite the huge number of nonzero elements, for its very large size, in real applications *M* cannot be stored and it must be recomputed whenever a matrix-vector product is needed.

We finally remark that we have modified the general approach presented in [40] by efficiently exploiting the characteristics of our specific mammographic setting. Really, since the DBT detector is a stationary flat panel and it is parallel to the compression plane of the breast, the footprints can be directly projected onto the detector plane, thus avoiding the use of an intermediate projection plane and further computational costs.

## 4. Materials

### 4.1. DBT System Configuration

Our tests are performed on the digital system *Giotto Class* of the Italian I.M.S. Giotto Spa company in Bologna [41]. The source executes $N_{\theta }=11$ scans from equally spaced angles in approximately 30 degrees range; in the highest vertical position, the source is about 70 cm over the detector. The stationary digital detector has a sensitive area of 24 cm × 30 cm and squared pixel pitch of 0.085 mm; the reconstructed voxel dimensions along the three Cartesian axes are $\Delta_{x}=\Delta_{y}=0.090$ mm and $\Delta_{z}=1$ mm, respectively.

The system uses a polychromatic ray with energies in a narrow range around 20 keV to avoid the photon scattering. As always happens in CT reconstruction algorithms, we approximate the polychromatic beam with a monochromatic one.

### 4.2. Data Sets

We consider two data sets in our experiments: a breast 3D phantom and a clinical acquisition from a human subject. Both volumes contain the objects of interest for breast cancer detection, i.e., small high contrast microcalcifications and larger but lower contrasted masses.

The phantom is the model 020 of BR3D breast imaging phantom, produced by CIRS Tissue Simulation and Phantom company [42]. It is characterized by a heterogeneous background, where adipose-like and gland-like tissues are mixed in about 50/50 ratio and it is made of six slabs that may be arranged to create multiple anatomical backgrounds. Each slab has a semicircular shape and its size is 10 cm × 18 cm. Inside one of them, we find acrylic spheres simulating breast masses (MSs), 1 cm length fibers and many clusters of calcium carbonate specks simulating microcalcifications (MCs). We report in Table 1 the length of the diameters of all the MSs and of each sphere of a MC cluster. In particular, we reconstruct a volume of 50 slices of 11.4 cm ×21 cm and we analyze in the reconstructed images objects with different diameters, such as the microcalcifications in clusters 3, 5 and 6 (having 230, 165 and 130 μm diameter, respectively) and the second and fourth mass (with diameter 4.7 and 3.1 mm, respectively). All such objects lie on the same slice.

As human DBT data set, we have chosen a case containing microcalcifications, circular and spiculated masses. The clinical volume is constituted of 55 slices of 10.5 cm × 20.7 cm.

### 4.3. Measure and Graphics of Merits

In order to quantitatively evaluate the reconstructed objects of interest in the volumes, we compute two widely used measure of merits: the Contrast-to-Noise Ratio (CNR) and the Full-Width at Half Maximum (FWHM).

The CNR measure on a mass is calculated as:

(24) $$CNR_{MS}=\frac{\mu_{MS}-\mu_{BG}}{\sigma_{MS}-\sigma_{BG}}$$

where μ and σ are the mean and standard deviation computed on the reconstructed volume, in small regions located inside the mass (MS) or in the background (BG). Similarly, we define the CNR measure on a microcalcification as:

(25) $$CNR_{MC}=\frac{M_{MC}-\mu_{BG}}{\sigma_{BG}}$$

where $M_{MC}$ is the maximum intensity inside the considered microcalcification (MC). Higher values of the CNR indices reflect a better detection of an object from the background.

To compute the FWHM parameter, we consider the transverse slice (parallel to the XY plane) where the microcalcification lies and we extract the *Plane Profile (PP)* along the *Y* axes. The FWMH index is computed as:

(26) $$FWHM=2\sqrt{2\ln\left(2\right)}d$$

where *d* is the standard deviation of the Gaussian curve fitting the PP. We remark that

(27) $$w=FWHM\cdot \Delta_{y}$$

approximates the width of the examined microcalcification. The Plane Profiles are also useful tools to evaluate the reconstruction accuracy on the transverse plane.

To estimate the solver effectiveness along the *Z* direction, which is the most challenging purpose in DBT imaging, we plot the Artifact Spread Function (ASF) vector, whose components are computed on a microcalcification as:

(28) $$ASF\left(z\right)=\frac{|\mu_{MC}\left(z\right)-\mu_{BG}\left(z\right)|}{|\mu_{MC}\left(\bar{z}\right)-\mu_{BG}\left(\bar{z}\right)|},\;\;\forall z=1,\ldots ,N_{z}$$

where μ(z) is the mean of the reconstructed values inside a circular region of three pixels diameter inside the considered MC and in the background, $\bar{z}$ corresponds to the slice where the object is on focus and $N_{z}$ is the total number of discrete slices. Similarly, we compute the ASF for the masses.

## 5. Numerical Results and Discussion

In this section we present the results obtained with the proposed optimization approach and the accurate solvers described in Section 3. At first we compare the BR3D phantom reconstructions produced by the SGP, FP and CP solvers in a similar computational time. Then, in order to analyze the best achievable image quality, we have run the SGP up to convergence both on the phantom and on the clinical data set. At last, we test the automatic rule proposed in Section 3.4 to decrease the λ values along the iterations.

When setting a constant value for λ is required, we fixed it by trial and error. The sequence ${\left\{\rho_{k}\right\}}_{k}$ for the SGP scheme has been defined as:

$$\rho_{k}=\sqrt{1+10^{15}/k^{2.1}}$$

as suggested in [31].

### 5.1. Methods Comparison for Early Reconstructions

The aim of this paragraph is to show the behavior of the proposed solvers at different stages of their executions. We fixed 5 and 15 iterations: the workload of 5 iterations is compatible with the execution of a reconstruction on a commercial hardware in a clinical setting, whereas in 15 iterations we get fairly accurate results with all the three methods, reflecting that they are sufficiently close to the computed convergence solution. From a computational perspective, the SGP and the CP iterations are almost equally expensive, hence we stopped them exactly at 5 or 15 iterations. In the special case of FP solver, the number of allowed iterations corresponds to the sum of the external and CG iterations: since we always perform 4 CG iterations, the FP execution is stopped after one or three outer iterations (loop *k* in Algorithm 2), respectively.

The value of β in (5) has been fixed as β=0.001. Since the three considered algorithms solve different optimization problems, the three λ parameters have been chosen independently for each method to achieve the best reconstruction in 5 iterations: we have set λ=0.005 for both SGP and CP methods and λ=0.001 for the FP algorithm.

**Figure 3:**
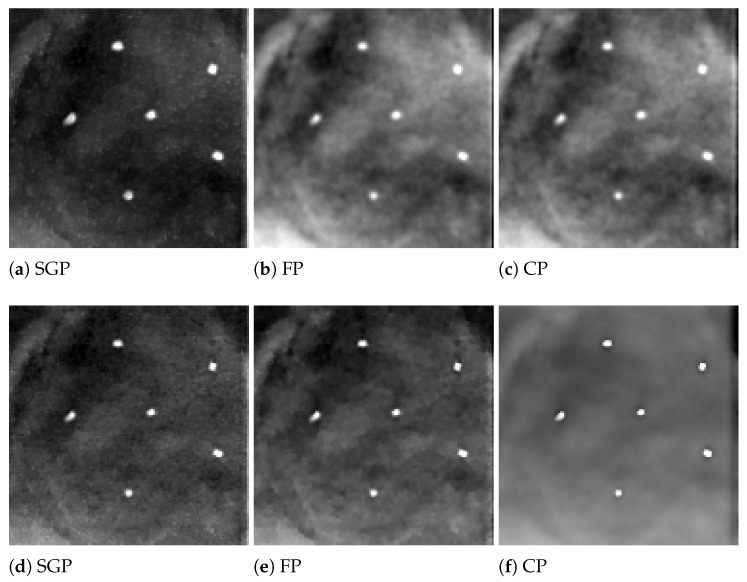
Reconstructions of microcalcification cluster number 3 in BR3D phantom obtained with Scaled Gradient Projection (SGP), Fixed Point (FP) and Chambolle–Pock (CP) methods. In the upper row, reconstructions in 5 iterations; in the bottom row, reconstructions in 15 iterations.

**Figure 4:**
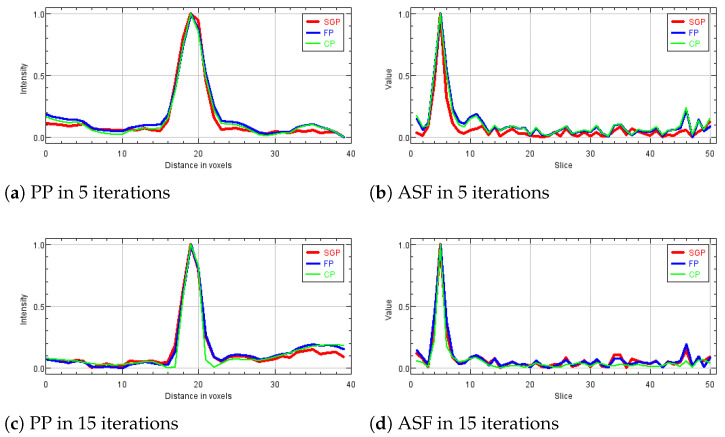
Plots of the Plane Profile on the left and of the Artifact Spread Function (ASF) vectors on the right, taken over one microcalcification of cluster number 3 in BR3D phantom obtained. In all the plots the red line corresponds to SGP method, the blue line to FP method and the green line to CP method.

In the following analysis, we focus on the reconstruction of MC cluster number 3 in the BR3D phantom. For each solver, Figure 3 reports a 125×125 pixels crop taken from the fifth slice of the reconstructions in 5 and 15 iterations. The images are represented by automatically enhancing the gray level contrast computed on the same considered region. In Figure 4 we compare the PP and ASF curves taken on one MC.

Looking at Figure 3, we observe that the detection of the MC cluster is comparable at equal iterations, whereas the background appears slightly different for the three methods. For example, in the case of CP reconstruction in 15 iterations it looks smoother and more blurred. Focusing on the objects of interest, we notice that in 5 iterations the MCs are perfectly visible; moreover, in 15 iterations the MC edges are sharper as confirmed by the plots (a) and (c) in Figure 4. From plots (b) and (d) of Figure 4, we observe that in all the three reconstructions the object is placed in the correct slice and it is not diffused in the adjacent layers. Hence, we can conclude that the proposed model-based optimization framework yields good quality images in early reconstructions, regardless the considered model formulation.

We highlight that the above results have been computed by a C-language-based scalar implementation of each solver, where it is not possible to reconstruct the whole volume of the phantom because of the too high memory demand. Working on one node HPE ProLiant DL560 Gen10 with 4 Intel(R) Xeon(R) Gold 6140 CPU @ 2.30 GHz and 512 Gb RAM, we considered cropped projections (corresponding to $4.5\cdot 10^{6}$ measurement data approximately) and we recovered a partial volume ($N_{v}\approx 5\cdot 10^{6}$ voxels). The execution time of the SGP, FP and CP functions resulted to be 262, 272 and 241 s, respectively, to perform 15 iterations. We remark that the reported time for the CP solver does not include the execution of step 1 since the approximation of Γ has been computed externally. As already mentioned, a parallel implementation is required to reconstruct a whole phantom volume or a real breast. The results reported in the following, have been achieved by a CUDA-based SGP software, exploiting one Nvidia Tesla V100 GPU board.

### 5.2. SGP Algorithm Insights

In the following, we raise the SGP as the representative solver in the proposed optimization framework. We explore the performance of the model-based approach on many different objects of the BR3D phantom (such as microcalcifications of very small diameter and masses with a low contrast with the background tissue) and we analyze the quality of the reconstructions also after 15 iterations, i.e., approaching convergence.

**Figure 5:**
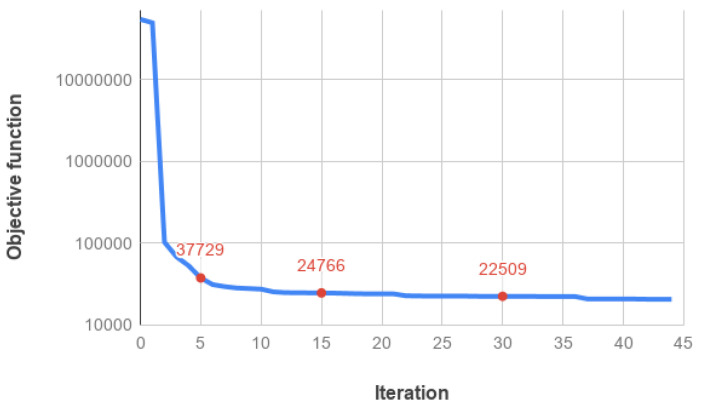
Objective function values vs. the iteration number for the SGP execution on the phantom test. The convergence has been reached after 44 iterations by satisfying condition (29). The red labels outline the function values at 5, 15 and 30 iterations.

In particular, we run the SGP solver on the BR3D phantom until the stopping condition:

(29) $$\left|\frac{f\left(x^{\left(k\right)}\right)-f\left(x^{\left(k-1\right)}\right)}{f(x^{\left(k\right)})}\right|<10^{-6}\;$$

is satisfied. It occurs after 44 iterations. In Figure 5, we plot the objective function values vs. the number of iterations: we observe that the objective function fast decreases in the first 5 iterations, whereas it exhibits a very flat trend from 10 iterations on, as it is confirmed by the red labeled values. We have seen, in fact, that the reconstructed images are visually almost indistinguishable after 30 iterations.

**Figure 6:**
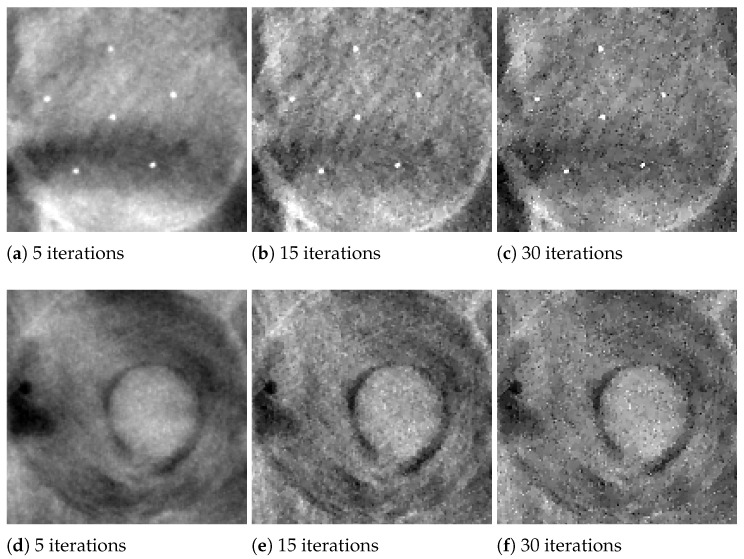
SGP results on BR3D phantom. (**a**–**c**) Reconstructions of MC cluster number 5 obtained after 5, 15 and 30 iterations. (**d**–**f**) Reconstructions of mass number 2 obtained after 5, 15 and 30 iterations.

**Figure 7:**
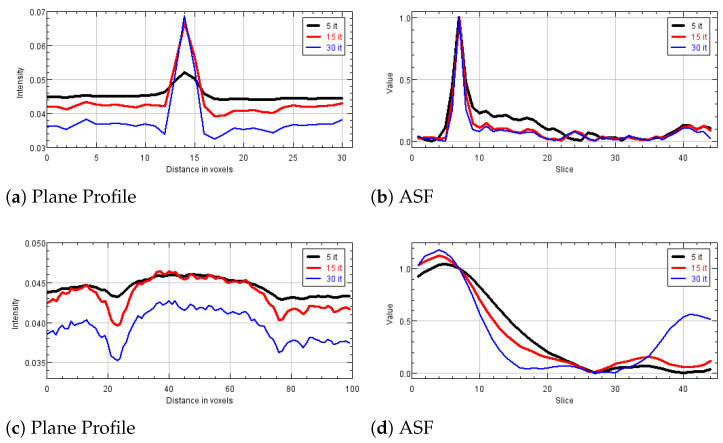
SGP results on BR3D phantom. Plots of the Plane Profile on the left and of the ASF vectors on the right, taken over one microcalcification of cluster 5 (**upper row**) and over mass number 2 (**bottom row**). In all the plots the black line corresponds to 5 iterations, red line to 15 iterations and blue line to 30 iterations.

In Figure 6, we exhibit the reconstructions of the 165 μm MCs of cluster 5, and the 4.7 mm mass (MS 2), obtained by the SGP algorithm after 5, 15 and 30 iterations. In Figure 7 we report the corresponding PP and ASF plots. Figure 6a shows that the MC of cluster 5 can be clearly visible after only 5 iterations and the PP plots of Figure 7a confirms that it gets more and more enhanced from the background. The ASF plot in Figure 7b shows an improvement in the object detection along the *Z* direction. As visible in Figure 6 and from the PP plot of Figure 7c, MS 2 is out of focus at 5 iterations but its contours become more and more defined when the algorithm approaches to convergence. The previous plots confirm that the proposed model with TV regularization is more effective in recovering high contrast objects such as microcalcifications than low absorbing structures such as masses.

In Table 2, we report the values of the CNR parameter on the examined reconstructed microcalcifications and masses. In particular, recalling the CNR definition (24) for the masses, the background area is a circle with diameter of 80 voxels, whereas we have considered circles of diameter 40 and 25 voxels inside the masses 2 and 4, respectively. When evaluating the CNR index on a MC with Equation (25), we regard as background area a circle of 20 voxels diameter and we compute M on a small circle of diameter 5 voxels containing the microcalcification.

Table 3 shows the values of the FWHM index defined in (26) and computed on one of the reconstructed microcalcification in each cluster. The corresponding MCs widths *w*, computed in micrometers as in (27), are reported for a comparison with the values of the actual diameters, shown in Table 1. Both tables demonstrate that we can get improved and more accurate reconstructions as the SGP approaches to the convergence: the increasing CNR indexes exhibit good denoising effects whereas the object enhancement is confirmed by the FWHM decreasing values. We remark that the MCs of cluster 6 are not discernible from the background in only 5 iterations (the FWHM is not measurable on the sixth MC cluster), because they are 130 μm width and they should approximately fill inside only two voxels. However, they can be well recovered in more iterations with a good approximation of their real size.

### 5.3. Experiments on a Human Data Set

**Figure 8:**
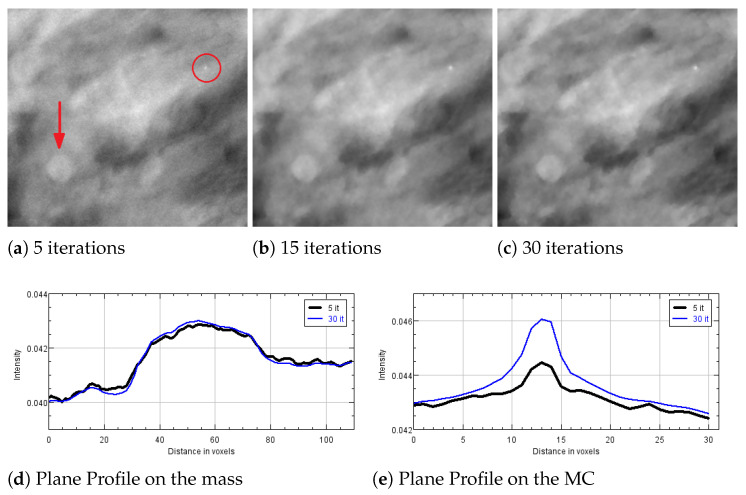
Results obtained after 5, 15 and 30 SGP iterations on a human breast data set. (**a**–**c**) Reconstructions of a 440 × 400 pixels region presenting both a spherical mass (pointed by the arrow) and a microcalficication (identified by the circle). (**d**,**e**) Plane profiles on the mass and on the microcalcification. In the plots: black line corresponds to 5 iterations and blue line to 30 iterations.

We now illustrate the results obtained by reconstructing a real breast volume with the SGP solver at different iterative stages. In Figure 8a–c, we report a crop of a reconstructed slice, where we can distinguish objects of interest, i.e., a spherical mass and a small microcalcification. The plots in Figure 8d,e represent the PP calculated on the mass and the microcalcification, respectively. The mass is well distinguishable since the earliest reconstruction and its shape and gray level intensity do not change remarkably; however the regular blue and thin profile in Figure 8d points out the denoising effects of the TV function in the last iterations. In addition, the microcalcification is detected in few iterations, even if a more time-consuming SGP execution enhances the contrast of the object with respect to the background. Table 4, reporting CNR and FWHM values computed on the objects in Figure 8, gives more insight on the quality of the reconstructions. In particular, it confirms that the noise progressively decreases and the microcalcification gets more and more defined, from 5 to 30 iterations.

**Figure 9:**
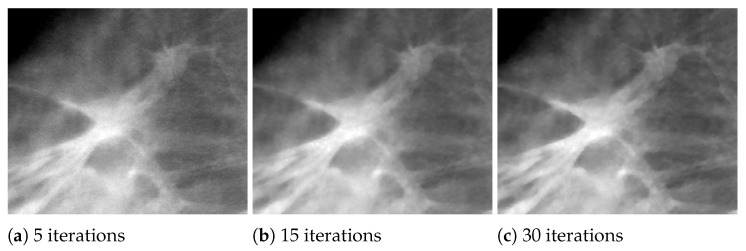
Results obtained after 5, 15 and 30 SGP iterations on a human breast data set. The reported 558 × 480 pixels crops present two speculated masses.

In Figure 9, we report the reconstruction of two speculated masses, which can occur in clinical cases. For such breast objects, the previous measures of merits are not applicable. However, we can observe that they both are well recognizable in the earliest reconstruction and the edges become sharper with increasing iterations.

### 5.4. Experiments with a Variable Regularization Parameter

In all the above experiments, we have set a constant value of the regularization parameter along the iterations, to let the solvers perform at their best on the prefixed model derived by the settled λ. In this paragraph, we show the results achieved with the automatic rule (23) for the choice of a decreasing sequence ${\left\{\lambda_{k}\right\}}_{k}$ applied to the SGP solver, to reconstruct the BR3D phantom.

**Figure 10:**
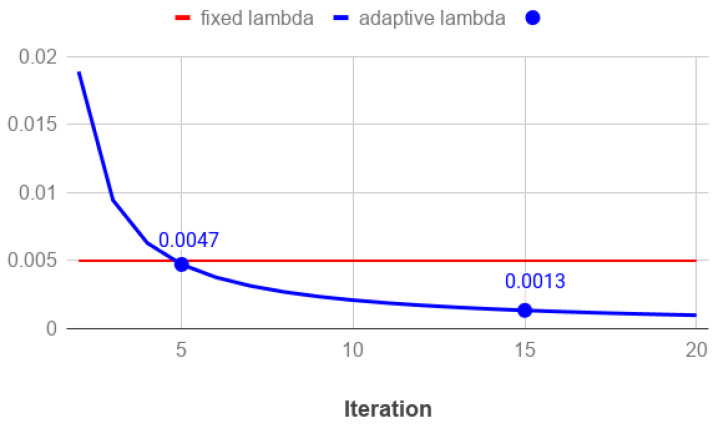
Plot of the set of values $\lambda_{k}$ versus the iteration *k* (blue line) in the SGP execution on the phantom test. The red straight line represents the constant value λ=0.005 used in SGP for the experiments presented in the previous sections.

**Figure 11:**
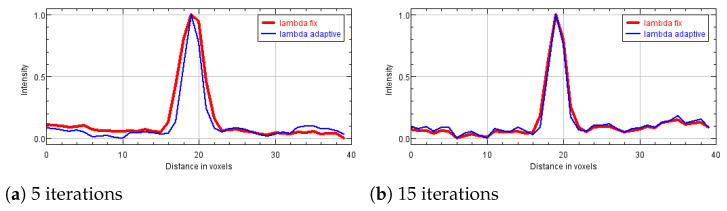
Plane Profiles on one microcalcification of cluster number 3 of the phantom, obtained with SGP with different regularization parameters, in 5 and 15 iterations. In all the plots the red line corresponds to fix parameter and the blue line to the adaptive choice of λ.

Figure 10 plots the sequence ${\left\{\lambda_{k}\right\}}_{k}$ with the blue line, while the red line represents the constant λ value used in the SGP implementation in the previous experiments. We observe that the proposed strategy computes values greater than the heuristically fixed one λ=0.005 until the fifth iteration. In Figure 11 we compare the PP of one microcalcification from cluster 3, reconstructed at 5 and 15 iterations using both a fixed value and the proposed strategy for the regularization parameter. We can infer from Figure 11a that the resulting larger TV weights in the first iterations produce more accurate results. However, on advanced reconstructions the differences are negligible. We can conclude that the proposed automatic strategy results very efficient.

## 6. Conclusions

In this paper, we have presented three model-based problem statements under a unique optimization framework for DBT image reconstruction. Each minimization problem has been solved by a convergent and efficient algorithm, for very large-size image reconstruction. We have also proposed a user-independent rule for selecting suitable values of the regularization parameter.

The results obtained with three solvers are encouraging. In early reconstructions, objects of interest of size greater than 150 μm are visible and correctly located in the volume, whereas the object detection quality improves and the noise drastically reduces if more iterations are allowed. When extending the computation from 5 to 30 algorithm iterations, the increasing rate of the CNR value lies in a range +150% to +280%. At last, we have shown that varying the regularization parameter along the iterations produces better results, especially in the early stage of the algorithm execution, when compared to the use of a fixed value heuristically chosen.

Since the three considered solvers produce comparable high quality reconstructions, we can conclude that the proposed model-based approach can be successfully used to detect the most interesting objects in an early diagnosis of breast tumor. 

## Figures and Tables

**Table 1:** Diameters of the microcalcifications (MC) and of the masses (MS) in the BR3D phantom as reported in [42]. Measures are in micrometers (m).

	1	2	3	4	5	6
MC	400	290	230	196	165	130
MS	6300	4700	3900	3100	2300	1800

**Table 2:** Values of the Contrast-to-Noise Ratio (CNR) index computed after 5, 15 and 30 SGP iterations. The CNR value computed on microcalcifications is defined as in (25), whereas the CNR value computed on the masses is defined as in (24).

	5 it.	15 it.	30 it.
MC cluster 3	24.21	33.34	38.00
MC cluster 5	10.03	19.00	28.00
MC cluster 6	7.27	11.02	17.00
MS 2	0.82	1.07	1.66
MS 4	0.87	1.00	1.33

**Table 3:** Full-Width at Half Maximum (FWHM) index (26) and *w* measures (27) computed on the reconstructed MCs of the BR3D phantom, after 5, 15 and 30 SGP iterations.

	FWHM			*w* (µm)		
	5 it.	15 it.	30 it.	5 it.	15 it.	30 it.
MC cluster 3	4.77	3.32	2.70	430	299	243
MC cluster 5	3.52	2.65	2.32	317	238	209
MC cluster 6	-	2.05	1.52	-	185	137

**Table 4:** Valuexs of the of CNR and FWHM indices computed on the mass and the microcalcification observable in Figure 8a–c.

	CNR			FWHM		
	5 it.	15 it.	30 it.	5 it.	15 it.	30 it.
MS	0.239	0.381	0.558	-	-	-
MC	8.78	16.59	16.49	8.57	7.81	7.29

## Data Availability

3rd Party Data.
